# Engineering the conserved and noncatalytic residues of a thermostable β-1,4-endoglucanase to improve specific activity and thermostability

**DOI:** 10.1038/s41598-018-21246-8

**Published:** 2018-02-13

**Authors:** Xiutao Chen, Weiguang Li, Peng Ji, Yang Zhao, Chengyao Hua, Chao Han

**Affiliations:** Shandong Key Laboratory for Agricultural Microbiology, College of Plant Protection, Shandong Agricultural University, Tai’an, Shandong 271018 China

## Abstract

Endoglucanases are increasingly applied in agricultural and industrial applications as a key biocatalyst for cellulose biodegradation. However, the low performance in extreme conditions seriously challenges the enzyme’s commercial utilization. To obtain endoglucanases with substantially improved activity and thermostability, structure-based rational design was carried out based on the *Chaetomium thermophilum* β-1,4-endoglucanase CTendo45. In this study, five mutant enzymes were constructed by substitution of conserved and noncatalytic residues using site-directed mutagenesis. Mutants were constitutively expressed in *Pichia pastoris*, purified, and ultimately tested for enzymatic characteristics. Two single mutants, Y30F and Y173F, increased the enzyme’s specific activity 1.35- and 1.87-fold using carboxymethylcellulose sodium (CMC-Na) as a substrate, respectively. Furthermore, CTendo45 and mutants exhibited higher activity towards β-D-glucan than that of CMC-Na, and activities of Y173F and Y30F were also increased obviously against β-D-glucan. In addition, Y173F significantly improved the enzyme’s heat resistance at 80 °C and 90 °C. More interestingly, the double mutant Y30F/Y173F obtained considerably higher stability at elevated temperatures but failed to inherit the increased catalytic efficiency of its single mutant counterparts. This work gives an initial insight into the biological function of conserved and noncatalytic residues of thermostable endoglucanases and proposes a feasible path for the improvement of enzyme redesign proposals.

## Introduction

Cellulose, the most abundant renewable carbon resource on earth, is generally considered a sustainable feedstock to replace fossil fuels for biochemical and biotechnological production^1^. As a strategy for cellulose utilization, enzymatic hydrolysis has been widely applied for practical applications^2–5^. Endoglucanase (EC 3.2.1.4), which randomly hydrolyses β-1,4-glucosidic bonds in amorphous regions of cellulose chains to catalyse the initial attack on the biopolymer, is a major catalyst for cellulose biodegradation^6,7^.

The high cost of preparation and low performance in extreme reaction conditions have been perceived as major bottlenecks to industrial applications^8–10^. One effective approach to reduce enzyme-related costs is to enhance enzyme performance^11^. Thus, recent developments in enzyme production focus on the improvement of hydrolysis efficiency and specific tolerability, allowing the production of cheaper and stronger enzymes for industrial use^12^. Rational protein engineering is an efficient genetic approach to optimize properties through structural analysis and functional prediction^13,14^. Recently, more attention has been drawn to the underlying function of important residues, along with practicality of modifying conserved noncatalytic residues^15–17^, to generate mutant enzymes with improved properties and to help elucidate structure-function relationships^18^.

Generally, thermostable enzymes have excellent tolerance to various harsh conditions, including high salt concentrations and extreme pHs^19,20^. More importantly, in order to profoundly improve hydrolysis efficiency at high temperatures while simultaneously reducing microbial contamination in reaction processes, it is important for enzymes to be both thermoactive and thermostable^21,22^. Therefore, thermostable endoglucanases with excellent activity at elevated temperatures are always preferred in commercial practice and are considered ideal candidates for enzyme engineering^7^.

In our previous work, a novel thermostable β-1,4-endoglucanase CTendo45 from *Chaetomium thermophilum*, which is a member of the glycoside hydrolase 45 family with high hydrolytic activity and good thermostability, was identified^23^. In this study, site-directed mutagenesis of conserved and noncatalytic residues of CTendo45 was implemented to further enhance specific activity and thermostability, providing a potential biocatalyst for industry.

## Results

### Homology modelling and construction of mutants

**Figure 1:**
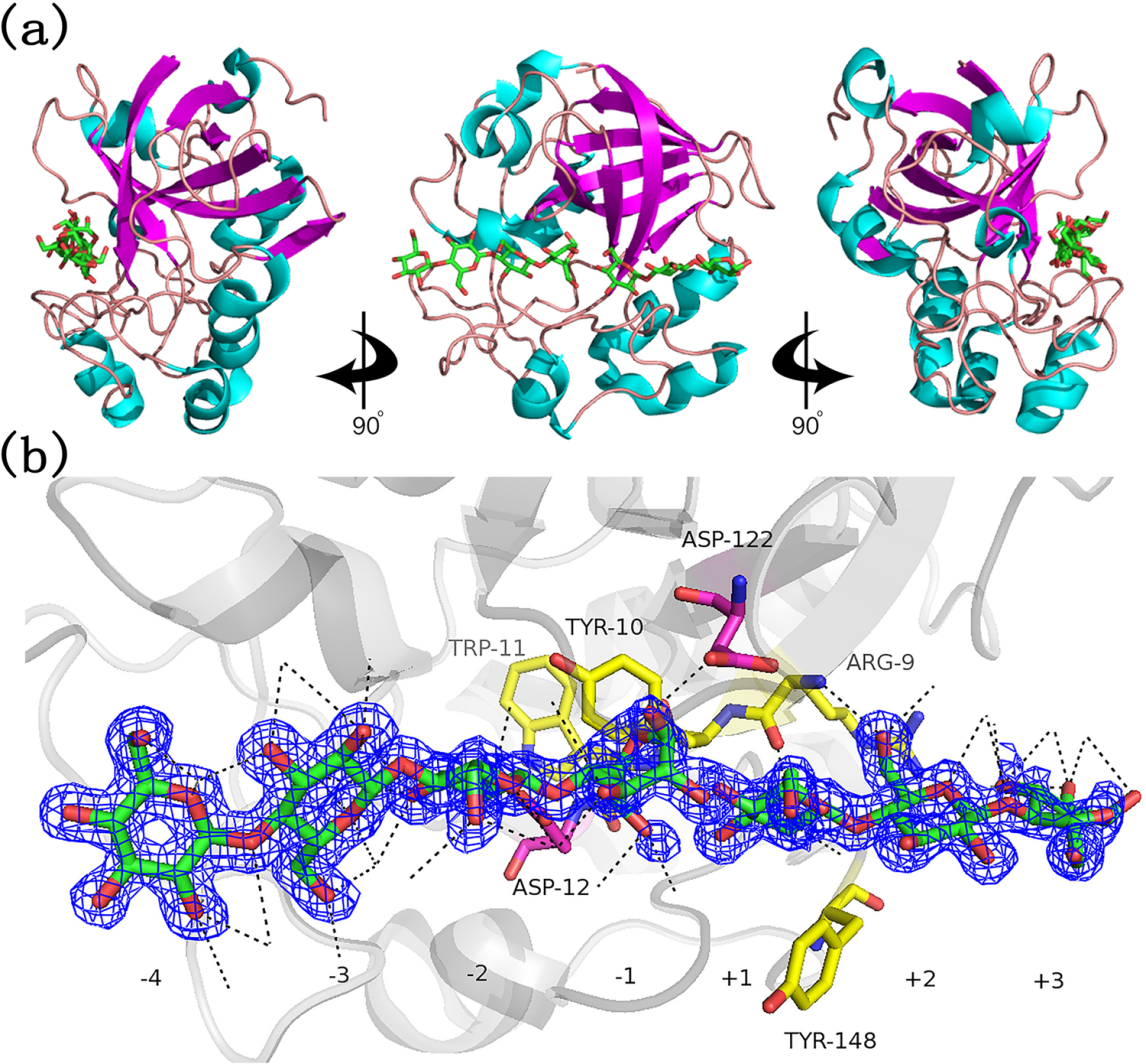
Structure of *Thielavia terrestris* β-1,4-endoglucanase TtCel45A (PDB: 5GLY). (**a**) Divergent stereo cartoon of TtCel45A structure in complex with cellotriose and cellotetraose molecules. (**b**) Observed electron density for ligands in the active site cleft. The yellow and purple sticks indicate the mutated noncatalytic residues and the catalytic residues, respectively. Hydrogen bonds are represented as black dashed lines. All of the structural diagrams were drawn using PyMOL software.

To improve specific activity and thermostability of the *C. thermophilum* endoglucanase CTendo45, rational protein engineering was carried out in this study. *Thielavia terrestris* β-1,4-endoglucanase TtCel45A (PDB: 5GLY) in complex with cellotriose and cellotetraose, carrying 64% amino-acid identity with CTendo45, was used as the basis for a homology model to predict the structure of candidate mutations^24^. As a typical GH45 endoglucanase^6,25^, TtCel45A exhibits the characteristic six-stranded β-barrel and a region consisting of several long interconnecting loops, which are partitioned by a substrate-binding cleft spanning the protein surface (Fig. 1a). The cleft is responsible for bringing the catalytic domain in an appropriate position for cellulose decomposition and gives space for seven glucose units (subsite −4 to +3) (Fig. 1b). In the cleft, Aspl22 is identified as a catalytic acid in the glycosyl group hydrolysis and Aspl2 acts as the base, enhancing the nucleophilicity of the catalytic water^25^. They are positioned above and to either side of a noncatalytic residue, Y10, which lies at the bottom of the active side groove^6^. The structural model also indicates the importance of Y148 based on its aromatic residue at the +1 subsite^24,26^. Detailed substrate interaction networks demonstrated that the main chains of R9 and W11 participate in the formation of subsites −1, +1 and +2^27,28^. It is therefore proposed that alterations of these noncatalytic residues which are located around the substrate binding site may reflect differences in the enzymatic properties and catalytic action^24,25^. Following the above rational, residues R29, Y30, W31 and Y173 in CTendo45, corresponding to residues R9, Y10, W11 and Y148 in TtCel45A, respectively, were chosen to determine the effects of mutations on enzyme characteristics.

R29 was substituted for H to make imidazole group replace guanido at that position in order to preserve the size and polarity of the side chain; Y30 and Y173 were substituted with F to determine the effect of hydroxyl removal from phenyl group; W31 was substituted with S to detect the result of replacing indolyl with hydroxyl at the side chain. Hence, four single mutants, R29H, Y30F, W31S and Y173F, were generated. Moreover, a double mutation Y30F/Y173F was constructed, depending on the hydrolytic activity of the corresponding single mutants. Modelled structures of mutants compared with the wild-type endoglucanase are exhibited in Supplementary Fig. S1.

### Production and purification of mutant enzymes

**Figure 2:**
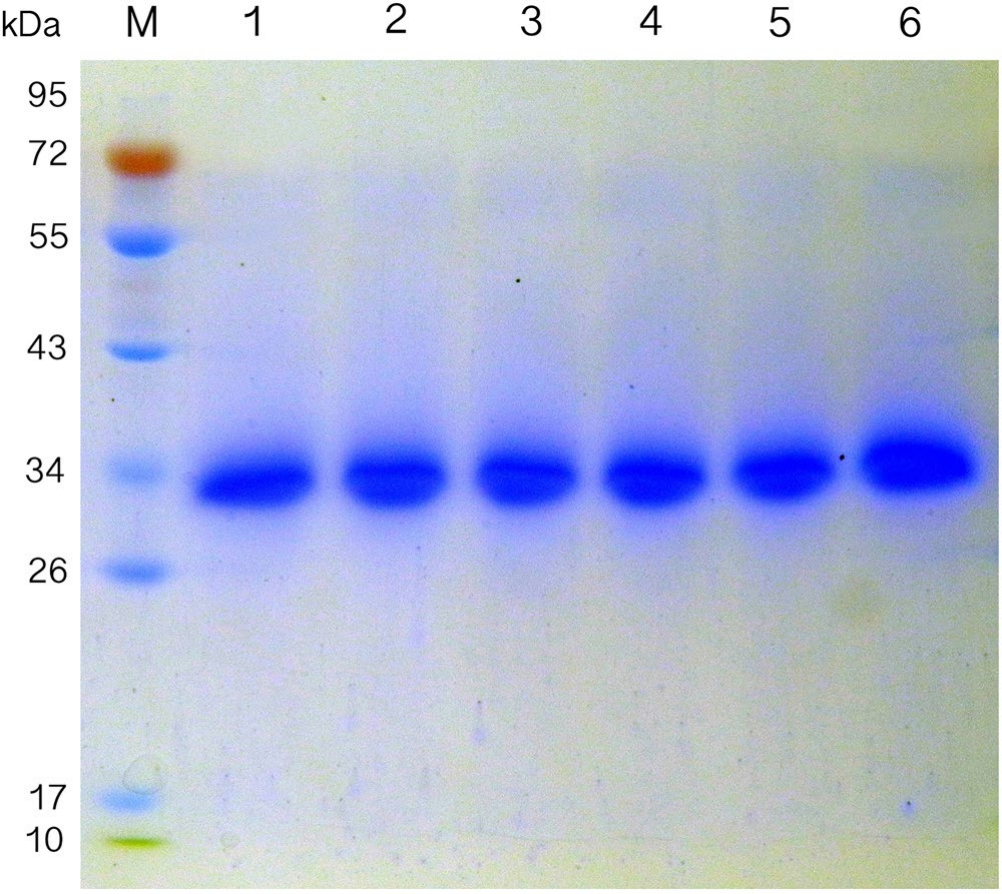
SDS-PAGE analysis of purified recombinant enzymes. Lane M, molecular mass markers; lane 1, the native CTendo45; lane 2, the R29H mutant; lane 3, the Y30F mutant; lane 4, the W31S mutant; lane 5, the Y173F mutant; lane 6, the Y30F/Y173F mutant. Digital image of gel is presented in Supplementary Fig. S2.

To investigate the enzymatic properties of these endoglucanases, CTendo45 and mutants were heterologously expressed in *P. pastoris* under the same condition and were then purified using Ni^2+^ affinity chromatography. The protein yield of each purified endoglucanase is shown in Table 1. SDS-PAGE gel electrophoresis indicated that each recombinant protein appeared as a single band with an approximate molecular mass of 32 kDa (Fig. 2; also see Supplementary Fig. S2).

**Table 1:** The protein yield of CTendo45 and mutants after Ni^2+^ affinity chromatographic purification. ^a^Values are the means ± SD of three replicates.

Enzyme	Protein yield (mg/L)^a^
CTendo45	358.9 ± 46.1
R29H	256.3 ± 27.5
Y30F	323.0 ± 55.1
W31S	344.6 ± 49.2
Y173F	407.8 ± 37.6
Y30F/Y173F	321.4 ± 39.7

### Optimum activity assay

**Figure 3:**
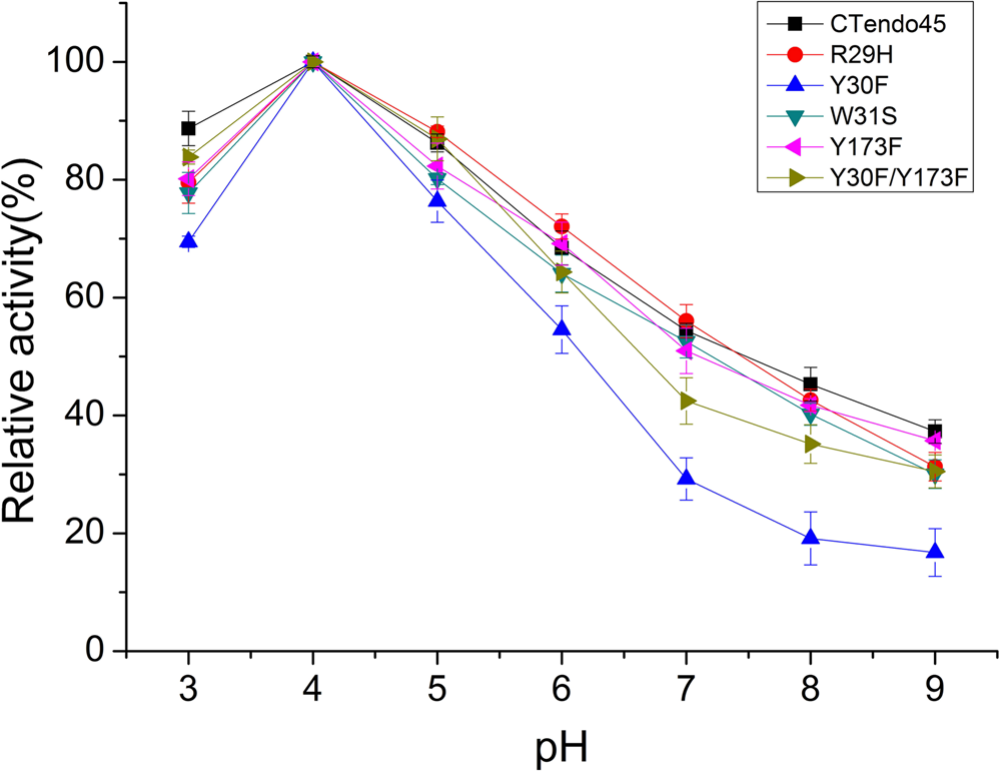
Optimal pH of mutant enzymes compared to the native CTendo45. The optimal pH value for each endoglucanase activity was assayed in 50 mM buffer solutions over a pH range from 3 to 9, including acetate buffer (pH 3–6), sodium phosphate buffer (pH 6–8) and Tris-HCl buffer (pH 8–9). The reaction was incubated at 60 °C for 30 min using 1% (w/v) CMC-Na as the substrate. The highest activity was defined as 100%. Values are the means ± SD of three replicates.

The optimum pH value for enzyme activity against sodium carboxymethyl cellulose (CMC-Na) was assayed in various buffer solutions in the pH range 3–9. As shown in Fig. 3, the optimum pH of all enzymes, including the wild-type endoglucanase and its mutants, showed no difference at pH 4 with relatively high activity in both neutral and acidic environments. Nevertheless, the activities of these enzymes were clearly reduced when the pH was above the optimum value, and relative activities were approximately 30% at pH 9 except for the mutant Y30F, which displayed a narrower pH profile and higher pH sensitivity compared to the other endoglucanases.

**Figure 4:**
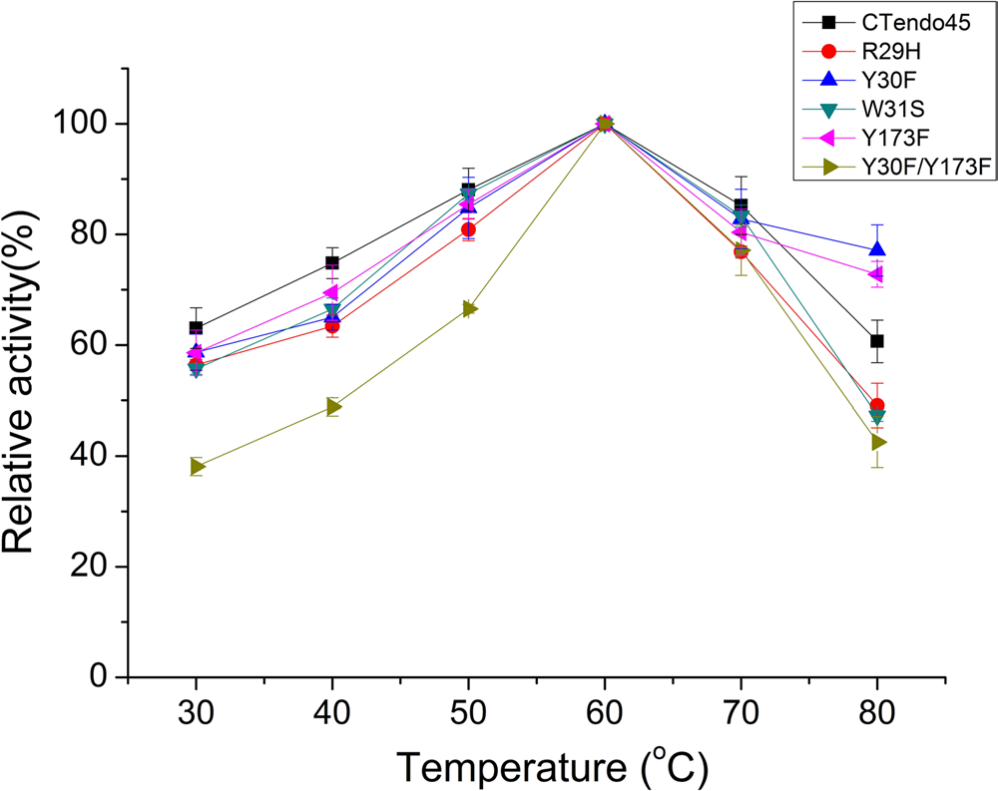
Optimal reaction temperature of mutant enzymes compared to the native CTendo45. The optimal reaction temperature for each endoglucanase activity was assayed at temperatures ranging from 30 °C to 80 °C in 50 mM acetate buffer (pH 4) using 1% (w/v) CMC-Na as the substrate. The highest activity was defined as 100%. Values are the means ± SD of three replicates.

The effect of temperature on enzyme activity against CMC-Na is shown in Fig. 4—all mutants had similar temperature optima to that of the wild type at 60 °C. The activities of the mutants R29H, W31S and Y30F/Y173F severely declined when the temperature exceeded 60 °C. For Y30F and Y173F, they maintained more than 70% of their maximal activity and showed higher relative activity than CTendo45 at 80 °C. These data indicate that the mutations of these selected conserved and noncatalytic residues had little impact on the enzyme’s optimum pH value and reaction temperature.

Similar results were observed using the native substrate of β-D-glucan, which showed no significant differences among these endoglucanases, at 60 °C and pH 4 (Supplementary Fig. S3).

### Specific activity and thermostability

The specific activity of purified endoglucanase mutants was detected on CMC-Na and β-D-glucan at the optimum reaction condition (Table 2). Compared with their wild-type counterpart, the activities of two single mutants, Y173F and Y30F, were increased 1.87-fold and 1.35-fold against CMC-Na, respectively. In addition, CTendo45 and mutants exhibited higher activity towards β-D-glucan than that of CMC-Na, and the hydrolysis activities of Y173F and Y30F were also increased obviously for β-D-glucan. However, it was unexpectedly observed that the activity of the double mutant Y30F/Y173F was reduced significantly, both for CMC-Na and β-D-glucan. Residue substitutions at either position R29 or W31 contributed to appreciably impaired activity; hence, the mutants R29H and W31S were not further analysed.

**Table 2:** Activities of CTendo45 and mutants on CMC-Na and β-D-glucan.

Substrate	Enzyme	Activity(IU/mg)^a^	Fold
CMC-Na	CTendo45	1.21 ± 0.08	1.00
	R29H	0.21 ± 0.03	0.17
	Y30F	1.63 ± 0.10	1.35
	W31S	0.44 ± 0.01	0.36
	Y173F	2.26 ± 0.04	1.87
	Y30F/Y173F	0.83 ± 0.05	0.68
β-D-glucan	CTendo45	2.07 ± 0.14	1.00
	R29H	0.46 ± 0.03	0.22
	Y30F	2.50 ± 0.13	1.21
	W31S	0.78 ± 0.04	0.38
	Y173F	3.46 ± 0.21	1.67
	Y30F/Y173F	0.94 ± 0.07	0.45

Each reaction was performed in 50 mM acetate buffer (pH 4) and incubation at 60 °C for 30 min using substrates of 1% CMC-Na and 0.2% β-D-glucan, respectively. ^a^Values are the means ± SD of three replicates.

**Figure 5:**
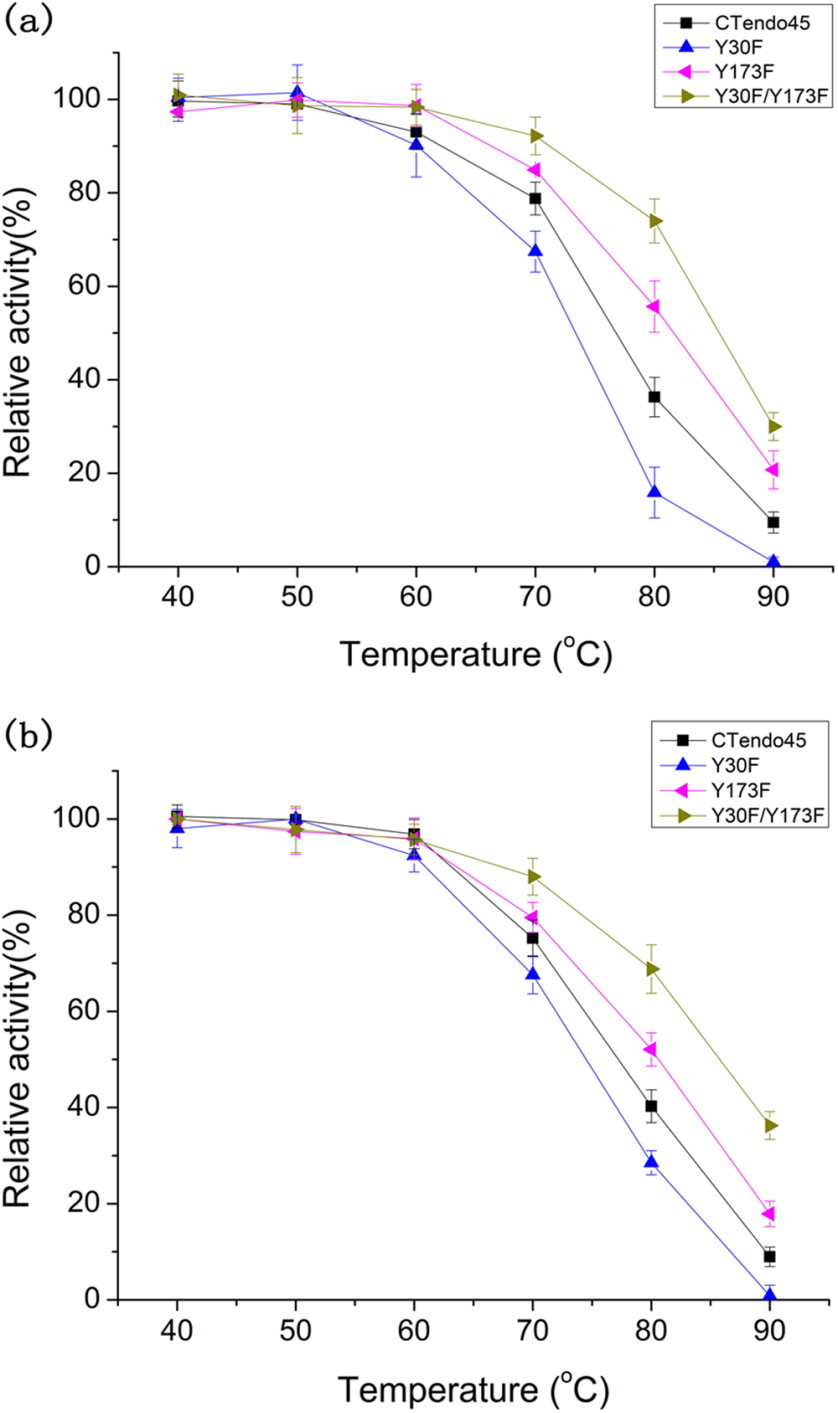
Thermostability of mutant enzymes compared to the native CTendo45. Enzymes were incubated for 200 min at different temperatures ranging from 40 °C to 90 °C before measurement of the remaining activity. The remaining activity of each endoglucanase was assayed at 60 °C for 30 min incubation using 1% (w/v) CMC-Na (**a**) and 0.2% (w/v) β-D-glucan (**b**) as substrates, respectively. The highest activity was defined as 100%. Values are the means ± SD of three replicates.

Based on the enzymatic activity data, three mutants (Y30F, Y173F and Y30F/Y173F) were selected to determine their thermostability using substrates of CMC-Na and β-D-glucan, respectively. After preincubation at different temperatures ranging from 40 °C to 90 °C for 200 min, the hydrolytic activities of these mutants were both reduced to varying degrees. Notably, after treatment at 80 °C and 90 °C for 200 min, the double mutant Y30F/Y173F exhibited excellent thermostability, retaining 73.9% and 29.1% activity against CMC-Na, respectively, and it exhibited relative insensitivity to high temperatures compared to the wild-type CTendo45 and other mutants (Fig. 5a). Consistent result of thermostability was obtained for Y30F/Y173F using β-D-glucan as a substrate, retaining 68.8% and 36.2% activity after heating at 80 °C and 90 °C for 200 min, respectively (Fig. 5b). For the single mutants, Y173F was more thermostable than CTendo45 at elevated temperatures, while Y30F nearly lost all activity at 90 °C after 200 min incubation with each substrate. The half-lives (t_1/2_) of these endoglucanases at 80 °C and 90 °C further demonstrated that their thermostability followed the order of Y30F/Y173F > Y173F > CTendo45 > Y30F (Table 3).

**Table 3:** Half-life of CTendo45 and mutants in thermal inactivation.

Substrate	Enzyme	t_1/2_ and residual relative activity	
		80 °C	90 °C
CMC-Na	CTendo45	150 min (48.8%)	35 min (52.9%)
	Y30F	120 min (46.2%)	20 min (54.1%)
	Y173F	210 min (48.9%)	50 min (53.0%)
	Y30F/Y173F	240 min (51.8%)	60 min (49.5%)
β-D-glucan	CTendo45	130 min (52.6%)	28 min (49.8%)
	Y30F	100 min (46.7%)	15 min (54.4%)
	Y173F	180 min (53.7%)	35 min (43.1%)
	Y30F/Y173F	220 min (48.4%)	45 min (46.5%)

Half-life (t_1/2_) was defined as the time that the enzyme activity declined to half of the initial activity value at temperatures of 80 °C and 90°, respectively. Untreated enzymes are considered as controls (100%). The residual relative activities are shown in brackets.

### Kinetic characterization of mutants

Michaelis-Menten kinetic constants for the wild-type CTendo45 and its site-directed mutants were determined with CMC-Na as the substrate. The reaction mixture was incubated for 30 min at the enzymes’ optimum condition of 60 °C and pH 4. The results are presented in Table 4. The Km values of all mutant enzymes showed increases compared to the wild type on CMC-Na. Nevertheless, the residue substitutions improved the reaction velocity, turnover rate and catalytic efficiency against CMC-Na. Among these mutants, Y173F caused the most significant effect as the kcat value was increased almost 4-fold. Additionally, the catalytic efficiency of Y173F was dramatically increased as the kcat/Km value was 1.94-fold higher than that of CTendo45. The Y30F mutation had no apparent effect on Km, while the kcat/Km value was increased 1.49-fold. Combining the two point mutations resulted in a 2.41-fold increase in Km value and lower catalytic efficiency than the other endoglucanases. These results indicated that the hydroxyl removal at each position, Y30 and Y173, effectively improved the catalytic efficiency on CMC-Na. Furthermore, it was revealed that simultaneous mutation of these two residues did not present an additive effect on catalytic efficiency. Kinetic parameters were also detected using β-D-glucan as the native substrate. The similar kcat/Km trend is shown in Table 4.

**Table 4:** Kinetic parameters of CTendo45 and mutants on CMC-Na and β-D-glucan.

Substrate	Enzyme	Km (mg/mL)^a^	Vmax (µg/min/mL)^a^	kcat (s^−1^)	kcat/Km (µL/s/mg)
CMC-Na	CTendo45	5.93 ± 0.54	4.42 ± 0.15	0.37 × 10^−3^	62.06
	Y30F	6.70 ± 0.66	7.45 ± 0.38	0.62 × 10^−3^	92.62
	Y173F	15.02 ± 0.65	18.10 ± 1.02	1.51 × 10^−3^	120.43
	Y30F/Y173F	14.29 ± 0.14	9.97 ± 0.85	0.83 × 10^−3^	58.14
β-D-glucan	CTendo45	2.08 ± 0.18	77.75 ± 6.58	6.27 × 10^−3^	3.02
	Y30F	0.85 ± 0.06	37.32 ± 4.18	3.24 × 10^−3^	3.80
	Y173F	0.99 ± 0.12	58.13 ± 6.37	5.23 × 10^−3^	5.27
	Y30F/Y173F	0.29 ± 0.04	9.80 ± 1.09	0.77 × 10^−3^	2.58

The reactions were performed in 50 mM acetate buffer (pH 4) at 60 °C for 30 min using a series of concentrations of CMC-Na or β-D-glucan (1 mg/mL to 10 mg/mL at the interval of 1 mg/mL) as the substrate with enzymes diluted to equivalent amounts. Kinetic parameters were calculated based on the Michaelis-Menten equation. ^a^Values are the means ± SD of three replicates.

## Discussion

Enzymatic hydrolysis of cellulose into fermentable sugars for subsequent processes is of considerable practical interest because of the tremendous application potential in the bioconversion of lignocellulosic biomass^8,29^. Although many endoglucanases of different sources have been isolated and commercialized^30,31^, more effective enzymatic properties were optimized to satisfy the industrial applications^32,33^. To obtain enzymes with higher thermostability and specific activity, rationally engineering based on the homologously modelled structure provides an effective strategy in the improvement of enzyme performance^34–37^.

In the present study, we employed a thermostable β-1,4-endoglucanase from *Chaetomium thermophilum* with high hydrolytic activity to construct mutants with improved specific activity and thermostability. Based on analysis of the corresponding homologous structure model (Fig. 1), four conserved and noncatalytic residues in the buried cleft around the substrate binding site were selected for site-directed mutagenesis (Fig. 1; also see Supplementary Fig. S4–S6). The wild type and mutant enzymes were successfully expressed in *P. pastoris* and then purified to determine enzymatic characteristics using CMC-Na and β-D-glucan, respectively (Fig. 2; also see Supplementary Fig. S2). As Figs 3 and  4 demonstrate, the wild type and mutant endoglucanases showed a similar pattern of their optimum reaction conditions, pH 4 and 60 °C, which was ascribed to there being no pronounced conformational rearrangements as a result of the residue substitutions^38^.

Two single mutants, Y30F and Y173F, significantly enhanced the hydrolytic activity, with a maximum increase of 1.87-fold and 1.67-fold against CMC-Na and β-D-glucan, respectively (Table 2). Moreover, the residue substitution at position Y173 improved the enzyme’s thermostability, while the mutant Y30F showed less temperature tolerance compared to the wild-type enzyme (Fig. 5 and Table 3). Residues Y30 and Y173 are located in the flexible loop close to the catalytic site (Fig. 1), so when the conserved tyrosine was replaced with phenylalanine, it would eliminate a hydrogen bond with the coordinated water molecule. This may initiate a moderate loosening of the buried cleft in the enzymatic structure, leading to functional improvement of catalytic residues^39^. Alternatively, changes in hydrogen bonding energy and the electronegative environment around the active site may also affect the specific activity^40^. The discrepancy of thermostability between Y30F and Y173F appeared to be associated with the amino acid positions located in different domains. The residue Y30 is closely situated near the six-stranded β-barrel, the backbone structure that preserves conformational stability; thus, slight conformational changes might interfere with the enzyme’s stability^36^, while the residue Y173 is located in the extended loop far from the β-barrel region (Supplementary Fig. S1). While elimination of the hydroxyl group of tyrosine in mutant enzymes contributes to a decrease in entropy and reduces sensitivity to elevated temperatures^41^, the introduction of phenylalanine at position Y30 can disrupt internal electrostatic interactions, which further affects structural stability^42^.

To determine whether simultaneous replacement of tyrosine by phenylalanine can enhance specific activity and thermostability, a double mutation, Y30F/Y173F, was designed. However, it was unexpectedly observed that Y30F/Y173F failed to increase the activity compared to its single-mutation counterparts. Surprisingly, as shown in Fig. 5 and Table 3, the resistance to high temperatures of Y30F/Y173F was much more remarkable than CTendo45 and even better than its single-mutation counterparts. These results demonstrated that double mutants do not always completely inherit the improved performance of their single-mutation counterparts, or at least not in a simple cumulative manner, which is analogous to previous studies^43–45^. The gain of extra thermostability would be caused by many complicated structural determinants that decrease the protein’s entropy, including hydrogen bond stabilization, compaction of secondary structure, and protein glycosylation^36,41,46^. However, we could not sufficiently illuminate the reason for the thermostability improvement and the activity decrease caused by Y30F/Y173F. More details regarding the mechanisms involved in the enhanced properties should be elucidated by resolving the 3D structure and investigating additional rational protein designs.

Further analysis of the kinetic parameters for CTendo45 and its mutant endoglucanases was carried out. CTendo45 is a typical member of GH45 endoglucanases and catalyzes polysaccharide hydrolysis via an inverting mechanism^23,24^, in which protonation of the glycosidic oxygen of residue Asp144 acted as a proton donor and aglycon departure is accompanied by a concomitant attack of a water molecule that is activated by the nucleophile/base residue Asp32^6,47,48^. Km values were increased for Y30F, Y173F and Y30F/Y173F at varying degrees using CMC-Na as a substrate, especially the mutant Y173F with a significant increase in kcat value, which may be concerned with a lower Km (Table 4). Replacement of a tyrosine side chain with a relatively smaller side group can weaken stacking interactions and result in a lower Km^36^. Since the kcat/Km ratio is the most informative feature for assessing the hydrogen bonding energy of the substrate, including the artificial substrate of CMC-Na and the native substrate of β-D-glucan, it can be used to explain the change in hydrolytic activity of the mutant enzymes^49^.

From an economic point of view, efficient catalytic activity is a very attractive enzyme property for practical applications^43^. Consequently, the Y173F mutant, which possessed improved thermostability and higher catalytic efficiency, can be a prospective candidate for wide biotechnological application.

## Conclusions

The biochemical characterization of five endoglucanase mutations based on CTendo45 indicated that the conserved and noncatalytic residues Y30 and Y173 play important roles in enzymatic properties, including thermostability, specific activity and kinetics. Noteworthily, it was demonstrated that one of the mutations, Y173F, prominently increased both specific activity and thermostability. Additionally, the mutation Y30F conferred an increase in activity but compromised thermostability. More interestingly, compared to the single-mutation counterparts and the original enzyme, the double mutant Y30F/Y173F enzyme exhibited enhanced resistance to elevated temperatures but appeared to have significantly less activity than we expected. This work gives an initial insight into the biological function of conserved and noncatalytic residues of thermostable endoglucanase and proposes a feasible path for the improvement of enzyme redesign proposals.

## Methods

### Strain, vector, and material

*Escherichia coli* T1 (TransGen Biotech, Beijing, China) was used for gene cloning. *Pichia pastoris* GS115 (Invitrogen, Carlsbad, CA, USA) was used for recombinant protein production as a heterologous expression host. The *Pichia-*secreted expression vector pPIC9K (Invitrogen, Carlsbad, CA, USA) was used for heterologous expression. The pPIC9K/*CTendo45* plasmid containing the β-1,4-endoglucanase gene *CTendo45* (GenBank accession number KC441877) and a C-terminal 6× histidine tag sequence was prepared as described previously^23^. The Fast Mutagenesis System Kit was purchased from TransGen Biotech (Beijing, China). Primers were synthesized by Sangon Biotech (Shanghai, China) and are listed in Supplementary Table S1. All chemicals were of reagent grade purity.

### Mutagenesis of CTendo45

Candidate mutation sites were selected according to crystal structural analysis of *Thielavia terrestris* β-1,4-endoglucanase TtCel45A, an enzyme highly homologous to CTendo45, in complex with cellotriose and cellotetraose (PDB: 5GLY)^24^. Different conserved and noncatalytic residues were selected to generate four single mutants (R29H, Y30F, W31S and Y173F) and one double mutant (Y30F/Y173F) (Supplementary Fig. S4). DNA sequences and translated amino acid sequences of CTendo45 and the designed mutants are shown in Supplementary Figs S5 and S6. Each mutant expression plasmid was individually generated by site-directed mutagenesis using PCR with pPIC9K/*CTendo45* plasmid as the template and then transformed into *E. coli* T1. Positive transformants containing mutant plasmids were screened on Luria-Bertani plates containing 50 µg/mL kanamycin after 14 h of incubation at 37 °C and then subsequently confirmed by DNA sequencing using self-primers and AOX1 gene primers (Supplementary Table S1). Correctly constructed recombinant plasmids were preserved and prepared for the next step.

### Transformation and heterologous expression in *Pichia pastoris*

Recombinant plasmids were individually *Sac*I-linearized and electroporated into *P. pastoris* GS115 cells^50^. Transformants that grew normally on MD and MM plates were seeded onto YPD medium plates supplemented with G418 (Sangon Biotech, Shanghai, China) at a final concentration of 1–4 mg/mL and cultured at 28 °C for three days to select multi-copy integrants. PCR amplification was carried out with the genomic DNA extracted from the selected multi-copy colony and AOX1 primers to confirm the presence of the mutant plasmid. Enzyme induction was performed under optimum shake-flask culture conditions at 28 °C according to the *Pichia* Yeast Expression System Kit (Invitrogen, Carlsbad, CA, USA)^51^.

### Purification and SDS-PAGE analysis

After induction by methanol for seven days, each crude culture was centrifuged at 8,000 rpm for 15 min to prepare a cell-free extract from the fermentation liquor. Then, the supernatant was collected and adjusted to 80% saturation with ammonium sulphate at 4 °C overnight. The suspension was centrifuged at 8,000 rpm for 15 min, and the precipitate was dissolved in phosphate buffer solution (pH 7.4). Histidine-tagged recombinant mutant enzymes were purified using Ni^2+^ affinity chromatography (HisTrap^TM^ FF crude; GE Healthcare, Buckinghamshire, UK)^23^. Protein concentrations were determined using a Pierce^TM^ BCA Protein Assay Kit (Thermo Scientific, Waltham, MA, USA). Molecular weights were confirmed by 12% (w/v) SDS-PAGE analysis using 15 µg protein for each enzyme.

### Activity assay

Sodium carboxymethyl cellulose (CMC-Na; Sigma-Aldrich, St. Louis, MO, USA) with a viscosity of 400–800 centipoise (cps) in water at room temperature and β-D-glucan from barley (Sigma-Aldrich, St. Louis, MO, USA) were used as substrates. The reaction mixture was composed of 150 µL of 1% (w/v) CMC-Na or 0.2% (w/v) β-D-glucan and 15 µg of purified enzyme in a 300 µL total reaction volume. The reaction was incubated for 30 min at 60 °C and then terminated by adding 300 µL of a 3,5-dinitrosalicylic acid reagent in a boiling water bath for 10 min^52^. After samples were cooled down to ambient temperature, the absorbance was measured at 540 nm. One international unit (IU) of enzyme activity was defined as the amount of enzyme that catalysed the liberation of reducing sugar equivalent to 1 μmoL of glucose per minute under the assay conditions^53^. Each experiment was performed in triplicate.

### Biochemical characterization of CTendo45 and its mutants

The optimal pH value for enzyme activity was determined in 50 mM buffer solutions over a broad pH range, including acetate buffer (pH 3–6), sodium phosphate buffer (pH 6–8) and Tris-HCl buffer (pH 8–9). The optimal temperature was evaluated at 30–80 °C at the optimal pH value. The relative hydrolytic activity was presented as a percentage of the released reducing sugar yield with the maximum of 100%^45^.

Thermostability was determined by assaying the residual activities after enzymes were preincubated at 40–90 °C for 200 min. Thermostability was assessed according to the ratio between residual activity and initial activity values. Moreover, the half-life (t_1/2_), which was defined as the time that the enzyme activity declined to half of the initial activity value at that temperature, was detected at 80 °C and 90 °C, respectively^43^.

### Kinetic characterization

The reaction was performed in 50 mM acetate buffer (pH 4) at 60 °C for 30 min using 1–10 mg/mL CMC-Na and β-D-glucan, respectively, with enzymes diluted to equivalent amounts. Kinetic parameters were calculated according to the Michaelis-Menten equation^42^.

### Availability of data and material

Recombinant strains described in this work are made available upon request to the corresponding author. Data sharing not applicable to this article as no datasets were generated or analysed during the current study.

## Electronic supplementary material

Supplementary information
