# Supplementary material for: Engineering the conserved and noncatalytic residues of a thermostable β-1,4-endoglucanase to improve specific activity and thermostability
Source: Sci Rep. 2018 Feb 13;8:2954. doi: 10.1038/s41598-018-21246-8 (PMC5811441; doi:10.1038/s41598-018-21246-8)
Supplement: Supplementary file 1 — Supplementary information [file 41598_2018_21246_MOESM1_ESM.pdf]

RESEARCH ARTICLE

# Engineering the conserved and noncatalytic residues of a thermostable β-1,4-endoglucanase to improve specific activity and thermostability

Xiutao Chen[+], Weiguang Li[+], Peng Ji, Yang Zhao, Chengyao Hua, and Chao Han*

Shandong Key Laboratory for Agricultural Microbiology, College of Plant Protection, Shandong Agricultural University, Tai'an, Shandong 271018, China

[+]these authors contributed equally to this work

SUPPLEMENTARY INFORMATION includes:

Supplementary Table S1

Supplementary Figures S1-S6

*Corresponding author. Address: Shandong Key Laboratory for Agricultural Microbiology, College of Plant Protection, Shandong Agricultural University, Tai'an, Shandong 271018, China.

*Email address*: hanch87@163.com (C.H.).

**Table S1.** Nucleotide sequences of primers used in this study.

| Primer | Sequence (from 5’ to 3’) |
|---|---|
| R29H-F | AGGCACCGGCAGAACAACCCACTACTGGGA |
| R29H-R | TGGGTTGTTCTGCCGGTGCCTTGGGCACCTT |
| Y30F-F | CACCGGCAGAACAACCCGCTTCTGGGATTG |
| Y30F-R | AAGCGGGTTGTTCTGCCGGTGCCTTGGGCA |
| W31S-F | CGGCAGAACAACCCGCTACTCGGATTGCTG |
| W31S-R | GAGTAGCGGGTTGTTCTGCCGGTGCCTTGG |
| Y173F-F | CAAACGGCTGGGGTGAGCGGTTCGGCGGG |
| Y173F-R | AACCGCTCACCCCAGCCGTTTGGAGGGGCG |
| 5’Self-primer | CAAGGTGCCCAAGGCA |
| 3’Self-primer | TTAGGGAGTCCAAGTCG |
| 5’AOX1 | GACTGGTTCCAATTGACAAGC |
| 3’AOX1 | GCAAATGGCATTCTGACATCC |

**Figure S1.** Structural comparison of mutants with the template of 5GLY created by SWISS-MODEL. Mutants are colored in golden and 5GLY is colored in purple.

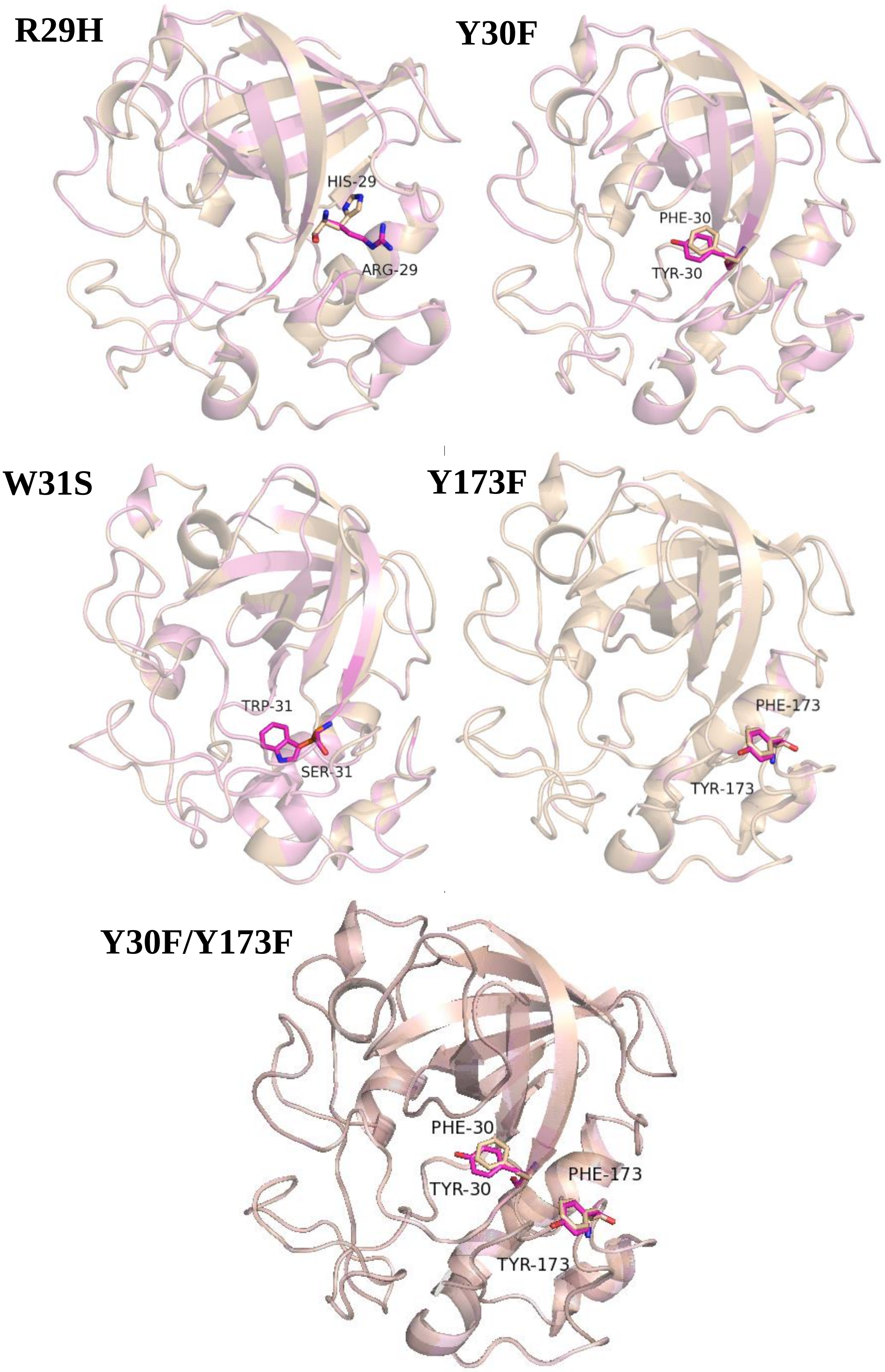

**Figure S2.** SDS-PAGE gel showing purified recombinant enzymes expressed in *Pichia pastoris*. lane 1, the native CTendo45; lane 2, the R29H mutant; lane 3, the Y30F mutant; lane 4, the W31S mutant; lane 5, the Y173F mutant; lane 6, the Y30F/Y173F mutant.

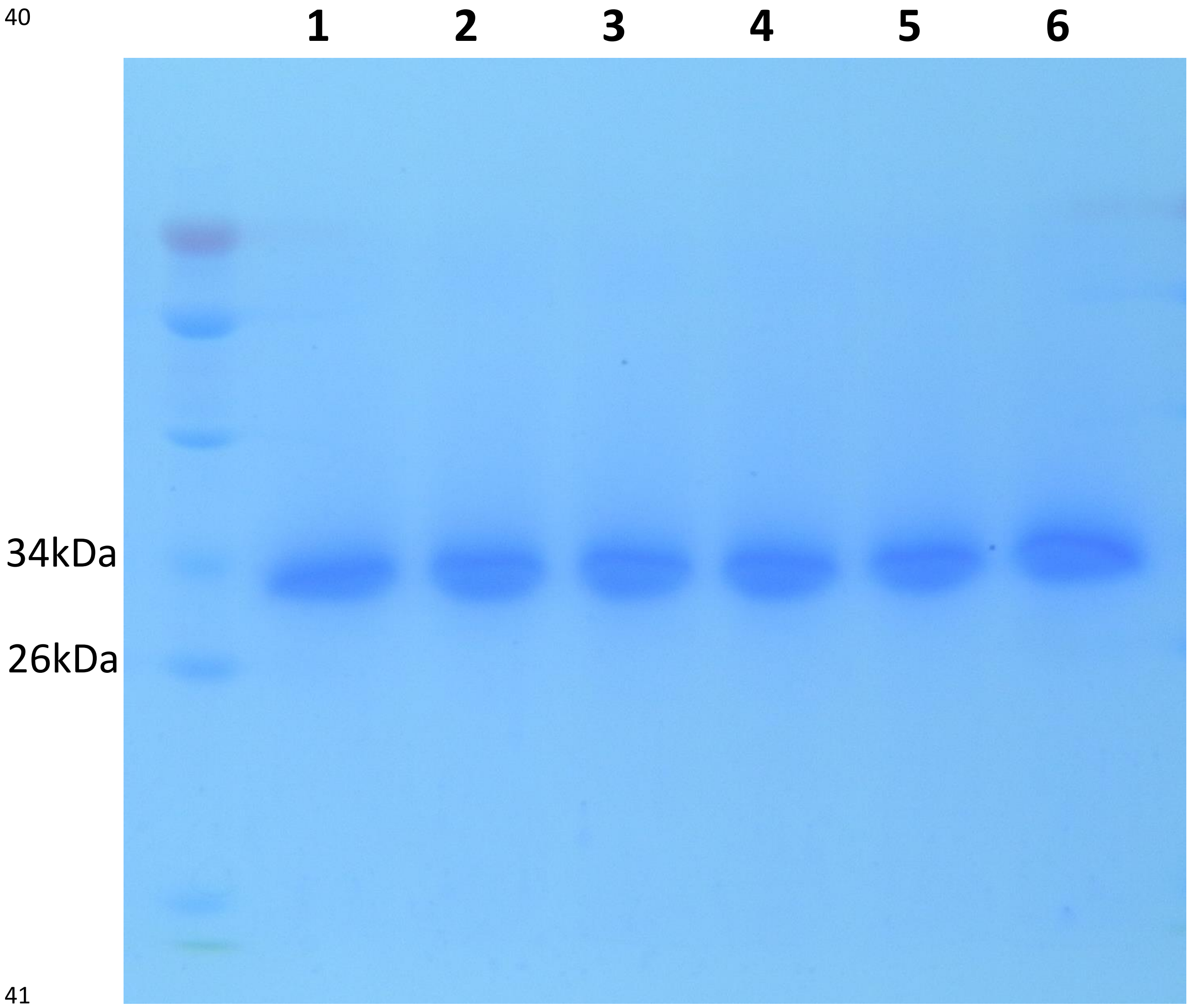

**Figure S3.** The optimal reaction pH (**A**) and temperature (**B**) of mutant enzymes compared to the native CTendo45. The optimal pH for each endoglucanase activity against 0.2% (w/v) β-D-glucan was assayed in 50 mM buffer solutions, including acetate buffer (pH 3-6), sodium phosphate buffer (pH 6-8) and Tris-HCl buffer (pH 8-9). The optimal reaction temperature for each endoglucanase activity was assayed at temperatures ranging from 30 ℃ to 80 ℃ in 50 mM acetate buffer (pH 4) using 0.2% (w/v) β-D-glucan as the substrate. The highest activity was defined as 100%. Values are the means ±SD of three replicates.

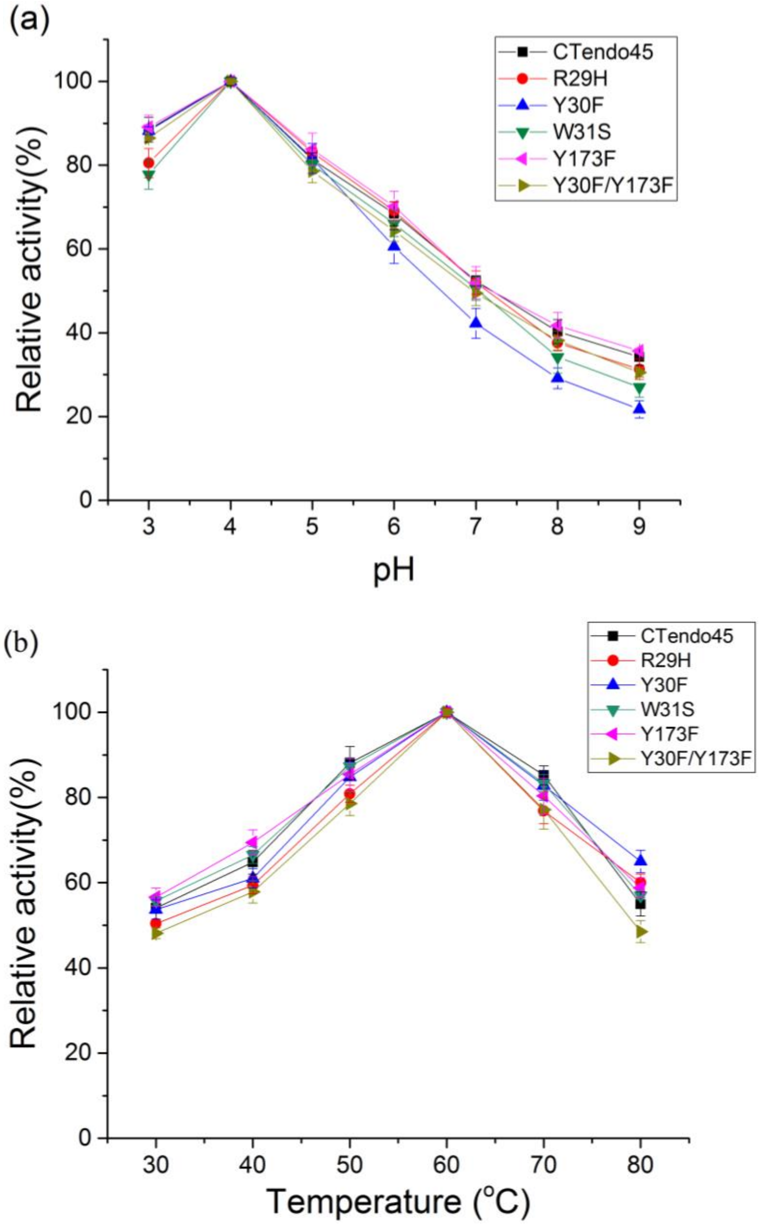

**Figure S4.** Sequence alignment of *C. thermophilium* CTendo45 with other GH45 endoglucanases. *Phaeoacremonium minimum* (XP_007916894), *Madurella mycetomatis* (KXX82926), *Myceliophthora thermophile* (XP_003659323), *Colletotrichum gloeosporioides* (EQB52129), *Colletotrichum tofieldiae* (KZL69663), *Humicola insolens* (PDB: 3ENG), *Melanocarpus albomyces* (PDB: 1OA7) and TtCel45A (PDB: 5GLY) from *Thielavia terrestris* using ClustalW2. Asterisk indicates the positions which have a single, fully conserved residue. Colon indicates the strongly similar parts among homologous sequences and period means the weakly similar parts among homologous sequences. Highlight blocks specify the catalytic residues Asp32 and Asp144 in CTendo45. The potential signal peptide is signed with black arrow. Different conserved and noncatalytic residues selected to generate mutants is noted by closed inverted triangle as R29, Y30, W31 and Y173 in CTendo45.

```
                               Signal peptide
M.myce      MHLPISTLITLLPFFLT--ATAQGPSGSGQTTRYWDCCKPSCAWPRKG--NSPSPVQTCD 56
P.mini      MQISLTALAALVPAYLAAVASAQGPSGSGQTTRYWDCCKPSCAWPGKGS-MSKGPLQTCS 59
CTendo45    MHLSQLAL----PLLLAAGAHAQGAQGTGRTTRYWDCCKPSCAWPGKS--NALQPVQTCN 54
C.gloe      MHPTQALVAL---FSLLPLAASQ-ASGTGSTTRYWDCCKPSCAWPGKAS-LAAGPVGTCD 55
C.tofi      MQPKQTLVSL---LPLLPFSTAQ-SSGTGSTTRYWDCCKPSCAWPGKSTQLASGPVTTCD 56
M.ther      MHLS-ATTGF---LALPALALAQ-LSGSGQTTRYWDCCKPSCAWPGKGP---SSPVQACD 52
H.inso      --------------------------ADGRSTRYWDCCKPSCGWAKKAP--VNQPVFSCN 32
M.albo      --------------------------ANGQSTRYWDCCKPSCGWRGKGP--VNQPVYSCD 32
5GLY        ------------------------ASGSGQSTRYWDCCKPSCAWPGKAA--VSQPVYACD 34
                                      . * :***********.*  *.      *: :*.

M.myce      RNDNPLNDGGNTRSGCDTGGSAYMCSSQSPWALNETVAYGWAAVNIAGSNEAAWCCSCYE 116
P.mini      KSDQPLNDGGSTKSGCDNGGGAFMCSNQSPWAVNETVSYGWAAVNIAGSNENSWCCACYE 119
CTendo45    AQDQPLNDGGNTRSGCDSGGSAFMCSNQSPWALNETLSYGWAAVRIAGQSEFNWCCACYE 114
C.gloe      RNDNRLNDGGATKSGCDAGGSAYMCSSNSPWAVSDSLAYGWAAVRISGGTEASWCCACYE 115
C.tofi      RNDNPLADGGATRSGCDSGGGAYMCSRNTPWAVSDDLAYGWAAVRISGGTEAQWCCACYE 116
M.ther      KNDNPLNDGGSTRSGCDAGGSAYMCSSQSPWAVSDELSYGWAAVKLAGSSESQWCCACYE 112
H.inso      ANFQRITD-FDAKSGCEPGGVAYSCADQTPWAVNDDFALGFAATSIAGSNEAGWCCACYE 91
M.albo      ANFQRIHD-FDAVSGCE-GGPAFSCADHSPWAINDNLSYGFAATALSGQTEESWCCACYA 90
5GLY        ANFQRLSD-FNVQSGCN-GGSAYSCADQTPWAVNDNLAYGFAATSIAGGSESSWCCACYA 92
             .  :  :  *    .  ***: ** *: *: ::***:.:  .: *:**. ::* .*  ***:**

M.myce      LTFTSGPVAGKKMIVQATNTGGDLGNNHFDIAMPGGGVGMFNACTNQYGAPPNGWGQRYG 176
P.mini      LTFTSGPVSGKKLIVQATNTGGDLGNNHFDLAIPGGGVGIFNACTSQYGAPSNGWGDRYG 179
CTendo45    LTFTSGPVAGKKMIVQATNTGGDLGSNHFDIAIPGGGVGIFNACTQQYGAPPNGWGERYG 174
C.gloe      LTFTSGPVSGKKMVVQATNTGGDLGQNHFDLAIPGGGVGQFNACTDQYGAPPNGWGQRYG 175
C.tofi      LTFTSGPVAGKKMIVQATNTGGDLGQNHFDIAIPGGGVGLFNACTDQYGAPANGWGQRYG 176
M.ther      LTFTSGPVAGKKMIVQATNTGGDLGDNHFDLAIPGGGVGIFNACTDQYGAPPNGWGDRYG 172
H.inso      LTFTSGPVAGKKMVVQSTSTGGDLGSNHFDLNIPGGGVGIFDGCTPQFGGLP---GQRYG 148
M.albo      LTFTSGPVAGKTMVVQSTSTGGDLGSNHFDLNIPGGGVGLFDGCTPQFGGLP---GARYG 147
5GLY        LTFTSGPVAGKTMVVQSTSTGGDLGSNQFDIAMPGGGVGIFNGCSSQFGGLP---GAQYG 149
            ********:**.::**:*.******.*:**:  :****** *:.*: *:*. .    * :**

M.myce      GIGSKSECEGFPAALKDGCNWRFDWFMGADNPDVTFRQVACPAAITAKSGCTRQNDVIDQ 236
P.mini      GIHSRSDCDSFPAALKAGCYWRFDWFGGADNPGVTFRQVACPAAITAKSGCVRNNDVINE 239
CTendo45    GIRSRSECDSFPEALKAGCYWRFDWFLGADNPDVSFKQVACPAAITAKSKCVRQRDVIDQ 234
C.gloe      GVSSRSECDGFPEKLKAGCYWRFDWFQGADNPSVSFKEVSCPAEITAKSGCRRN------ 229
C.tofi      GISSRSECDGFPEKLKAGCYWRFDWFKGADNPAVSFKQVTCPAEITNKSGCRRQG----- 231
M.ther      GIHSKEECESFPEALKPGCNWRFDWFQNADNPSVTFQEVACPSELTSKSGCSR------- 225
H.inso      GISSRNECDRFPDALKPGCYWRFDWFKNADNPSFSFRQVQCPAELVARTGCRRNDDGNFP 208
M.albo      GISSRQECDSFPEPLKPGCQWRFDWFQNADNPSFTFERVQCPEELVARTGCRRHDDGGFA 207
5GLY        GISSRDQCDSFPAPLKPGCQWRFDWFQNADNPTFTFQQVQCPAEIVARSGCKRNDDSSFP 209
            *: *:.:*: **   ** ** ****** .**** .:*..* **  :.  :: * *

M.myce      TPTGPSTVPTWTP-- 249
P.mini      TPTGESSTPTWTPSG 254
CTendo45    TPTGPEIVPTWTP-- 247
C.gloe      ---------------
C.tofi      ---------------
M.ther      ---------------
H.inso      AVQIP---------- 213
M.albo      VFKAPSA-------- 214
5GLY        VFTP----------- 213
```

**Figure S5.** DNA sequences of CTendo45 and designed mutants.

```
CTendo45    ATGCATCTCTCTCAGCTTGCCCTCCCCTTGCTCCTCGCTGCGGGTGCTCA 50
R29F        -------------------------------------------------- 50
Y30F        -------------------------------------------------- 50
W31S        -------------------------------------------------- 50
Y173F       -------------------------------------------------- 50
Y30F/Y173F  -------------------------------------------------- 50

CTendo45    CGCCCAAGGTGCCCAAGGCACCGGCAGAACAACCCGCTACTGGGATTGCT 100
R29F        -----------------------------------a-------------- 100
Y30F        --------------------------------------t----------- 100
W31S        -----------------------------------------c-------- 100
Y173F       -------------------------------------------------- 100
Y30F/Y173F  --------------------------------------t----------- 100

CTendo45    GTAAGCCCTCATGCGCCTGGCCCGGAAAGTCCAACGCCCTGCAACCAGTG 150
R29F        -------------------------------------------------- 150
Y30F        -------------------------------------------------- 150
W31S        -------------------------------------------------- 150
Y173F       -------------------------------------------------- 150
Y30F/Y173F  -------------------------------------------------- 150

CTendo45    CAAACTTGCAATGCGCAGGACCAGCCCCTGAACGATGGGGGCAACACGCG 200
R29F        -------------------------------------------------- 200
Y30F        -------------------------------------------------- 200
W31S        -------------------------------------------------- 200
Y173F       -------------------------------------------------- 200
Y30F/Y173F  -------------------------------------------------- 200

CTendo45    CTCCGGCTGCGACTCGGGCGGCAGCGCTTTCATGTGCTCAAACCAATCGC 250
R29F        -------------------------------------------------- 250
Y30F        -------------------------------------------------- 250
W31S        -------------------------------------------------- 250
Y173F       -------------------------------------------------- 250
Y30F/Y173F  -------------------------------------------------- 250

CTendo45    CCTGGGCGCTGAACGAGACACTCTCGTACGGCTGGGCGGCGGTTAGGATC 300
R29F        -------------------------------------------------- 300
Y30F        -------------------------------------------------- 300
W31S        -------------------------------------------------- 300
Y173F       -------------------------------------------------- 300
Y30F/Y173F  -------------------------------------------------- 300
```

```
CTendo45    GCGGGCCAGAGTGAATTCAACTGGTGCTGTGCGTGTTATGAATTGACTTT 350
R29F        -------------------------------------------------- 350
Y30F        -------------------------------------------------- 350
W31S        -------------------------------------------------- 350
Y173F       -------------------------------------------------- 350
Y30F/Y173F  -------------------------------------------------- 350

CTendo45    TACCAGTGGGCCGGTGGCGGGGAAGAAGATGATTGTGCAAGCGACGAATA 400
R29F        -------------------------------------------------- 400
Y30F        -------------------------------------------------- 400
W31S        -------------------------------------------------- 400
Y173F       -------------------------------------------------- 400
Y30F/Y173F  -------------------------------------------------- 400

CTendo45    CGGGCGGGGATTTGGGGAGTAATCATTTTGATATTGCTATCCCTGGTGGT 450
R29F        -------------------------------------------------- 450
Y30F        -------------------------------------------------- 450
W31S        -------------------------------------------------- 450
Y173F       -------------------------------------------------- 450
Y30F/Y173F  -------------------------------------------------- 450

CTendo45    GGTGTTGGTATCTTCAATGCCTGCACCCAACAATACGGCGCCCCTCCAAA 500
R29F        -------------------------------------------------- 500
Y30F        -------------------------------------------------- 500
W31S        -------------------------------------------------- 500
Y173F       -------------------------------------------------- 500
Y30F/Y173F  -------------------------------------------------- 500

CTendo45    CGGCTGGGGTGAGCGGTACGGCGGGATCCGCTCGCGCAGCGAGTGCGACA 550
R29F        -------------------------------------------------- 550
Y30F        -------------------------------------------------- 550
W31S        -------------------------------------------------- 550
Y173F       -----------------t-------------------------------- 550
Y30F/Y173F  -----------------t-------------------------------- 550

CTendo45    GCTTCCCCGAGGCGCTCAAAGCCGGCTGCTACTGGCGTTTCGACTGGTTC 600
R29F        -------------------------------------------------- 600
Y30F        -------------------------------------------------- 600
W31S        -------------------------------------------------- 600
Y173F       -------------------------------------------------- 600
Y30F/Y173F  -------------------------------------------------- 600
```

```
CTendo45    CTGGGTGCCGACAACCCGGACGTCTCTTTCAAGCAGGTGGCTTGCCCGGC 650
R29F        -------------------------------------------------- 650
Y30F        -------------------------------------------------- 650
W31S        -------------------------------------------------- 650
Y173F       -------------------------------------------------- 650
Y30F/Y173F  -------------------------------------------------- 650

CTendo45    AGCCATCACGGCCAAGAGCAAGTGCGTGCGACAGCGGGATGTCATCGACC 700
R29F        -------------------------------------------------- 700
Y30F        -------------------------------------------------- 700
W31S        -------------------------------------------------- 700
Y173F       -------------------------------------------------- 700
Y30F/Y173F  -------------------------------------------------- 700

CTendo45    AGACGCCGACTGGACCGGAGATTGTCCCGACTTGGACTCCCTAA 744
R29F        -------------------------------------------- 744
Y30F        -------------------------------------------- 744
W31S        -------------------------------------------- 744
Y173F       -------------------------------------------- 744
Y30F/Y173F  -------------------------------------------- 744
```

**Figure S6.** Translated amino acids sequences of CTendo45 and designed mutants.

```
CTendo45    MHLSQLALPLLLAAGAHAQGAQGTGRTTRYWDCCKPSCAWPGKSNALQPV 50
R29H        ----------------------------h--------------------- 50
Y30F        -----------------------------f-------------------- 50
W31S        ------------------------------s------------------- 50
Y173F       -------------------------------------------------- 50
Y30F/Y173F  -----------------------------f-------------------- 50

CTendo45    QTCNAQDQPLNDGGNTRSGCDSGGSAFMCSNQSPWALNETLSYGWAAVRI 100
R29H        -------------------------------------------------- 100
Y30F        -------------------------------------------------- 100
W31S        -------------------------------------------------- 100
Y173F       -------------------------------------------------- 100
Y30F/Y173F  -------------------------------------------------- 100

CTendo45    AGQSEFNWCCACYELTFTSGPVAGKKMIVQATNTGGDLGSNHFDIAIPGG 150
R29H        -------------------------------------------------- 150
Y30F        -------------------------------------------------- 150
W31S        -------------------------------------------------- 150
Y173F       -------------------------------------------------- 150
Y30F/Y173F  -------------------------------------------------- 150

CTendo45    GVGIFNACTQQYGAPPNGWGERYGGIRSRSECDSFPEALKAGCYWRFDWF 200
R29H        -------------------------------------------------- 200
Y30F        -------------------------------------------------- 200
W31S        -------------------------------------------------- 200
Y173F       ----------------------f--------------------------- 200
Y30F/Y173F  ----------------------f--------------------------- 200

CTendo45    MHLSQLALPLLLAAGAHAQGAQGTGRTTRYWDCCKPSCAWPGKSNAL 247
R29H        ----------------------------------------------- 247
Y30F        ----------------------------------------------- 247
W31S        ----------------------------------------------- 247
Y173F       ----------------------------------------------- 247
Y30F/Y173F  ----------------------------------------------- 247
```
